# Adherence to oxidative balance scores and lower odds of non-alcoholic fatty liver disease: a case–control study

**DOI:** 10.1038/s41598-023-33407-5

**Published:** 2023-04-15

**Authors:** Mohammad Hassan Sohouli, Pejman Rohani, Mahdieh Hosseinzadeh, Azita Hekmatdoost

**Affiliations:** 1 Student Research Committee, Department of Clinical Nutrition and Dietetics, Faculty of Nutrition and Food Technology, Shahid Beheshti University of Medical Sciences, Tehran, Iran; 2 Pediatric Gastroenterology and Hepatology Research Center, Pediatrics Centre of Excellence, Children’s Medical Center, Tehran University of Medical Sciences, Tehran, Iran; 3 Department of Nutrition, School of Public Health, Shahid Sadoughi University of Medical Sciences, Yazd, Iran; 4 Department of Clinical Nutrition and Dietetics, Faculty of Nutrition and Food Technology, National Nutrition and Food Technology Research Institute Shahid Beheshti University of Medical Sciences, No 7, West Arghavan St, Farahzadi Blvd, PO Box 19395-4741, Tehran, 1981619573 Iran

**Keywords:** Gastrointestinal diseases, Nutrition disorders, Risk factors

## Abstract

Evidence has also shown that oxidative stress and systemic inflammation, or in other words, disruption of the oxidant and antioxidant balance, can play an important role in the initiation or progression of NAFLD. The purpose of this study was to investigate the associations between the oxidative balance scores (OBS) and the risk of NAFLD. 552 healthy and 340 patients adult over the age of 18 with NAFLD participated in this case–control research. A validated 168-item quantitative food frequency questionnaire (FFQ) and indicators of physical activity, obesity, and smoking status were used to assess OBS score. The connection between OBS and NAFLD was discovered using binary logistic regression. The mean (± SD) age and (body mass index) BMI of the study population was 40.22 ± 9.79 years and 29.06 ± 3.92 kg/m^2^, respectively. The mean ± SD of OBS was 41.48 ± 5.23. After adjustment for potential confounders, higher scores of adherence to the OBS conferred a protection for the presence of NAFLD (odds ratio [OR]: 0.29; 95% confidence interval [CI]: 0.15–0.49; P for trend < 0.001). The findings of the present study indicate an approximately 80% reduction in the odds of developing NAFLD with higher OBS adherence in the overall population*.* However, prospective studies are needed to further investigate this association.

## Introduction

Non-alcoholic fatty liver disease (NAFLD) is becoming the most common form of chronic liver disease; it is characterized by a wide range of fat liver illnesses that can result in severe liver disease and cirrhosis^1^. The worldwide prevalence of NAFLD in adults is estimated to be at 20–25, 5–18, and 25–31% in populations in Asian countries and Iran, respectively^2–4^. NAFLD creates a significant economic burden on the health care system and affects the quality of life as the illness advances; thus, it is crucial to find effective solutions to prevent and treat this condition^5^. This high frequency of NAFLD is assumed to be mostly related to poor dietary habits, notably the consumption of a high-calorie diet that is rich in saturated fatty acids (SFA) or simple carbohydrates^6^. Regarding the pharmacological options of NAFLD, there is currently no consensus. Nonetheless, lifestyle therapies focusing on physical activity and well-balanced diet in terms of both quality and quantity are regarded as the cornerstone of NAFLD management^1^. Thus, a viable strategy for the prevention and treatment of NAFLD may involve alterations to eating habits and diet composition.

On the other hand, scientists have been interested in a number of diet-related risk factors that appear to contribute to NAFLD, including obesity, insulin resistance, inflammation, and oxidative and antioxidant systems^7–9^. Despite earlier research indicating a relationship between particular nutrients/food with oxidative stress and inflammation, dietary interactions may affect the effects of combined nutrients^10,11^. As a result, a novel method based on dietary patterns connected to oxidative and antioxidant systems is required.

Evidence has also shown that oxidative stress and systemic inflammation, or in other words, disruption of the oxidant and antioxidant balance, can play an important role in the initiation or progression of NAFLD through increasing lipid peroxidation in the cell membrane^12,13^.

Oxidative balance score (OBS) was first introduced by van Hooydonk et al. in 2002^14^ as a measure of antioxidant-prooxidant status, which included vitamin C and beta-carotene as antioxidant components and iron as a prooxidant factor. However, in later research and evidence, these dietary index components increased to 20^15–17^. In general, the components of OBS include two components of diet and lifestyle, which are categorized as antioxidant and prooxidant factors^18^.

Recent studies also show the significant and beneficial effect of antioxidant agents such as vitamin E and C on the development and even the onset of NAFLD disease^19–21^. In addition, there are various evidences for the role of various types of prooxidants and oxidative stress, such as increased intake of saturated fatty acids, obesity and smoking, on increasing the risk of this disease^19,22–27^.

Although some research has been done to correlate this index with the risk of chronic illnesses such as diabetes, cancer, and metabolic syndrome, no research has been done in NAFLD patients. On the other hand, due to the rising prevalence of NAFLD and the fact that chronic diseases, particularly NAFLD, have imposed significant costs on the health-care system, we conducted this study to assess OBS in NAFLD patients in order to identify a critical approach to improving or controlling NAFLD.

## Methods

### Study design and population

**Figure 1:**
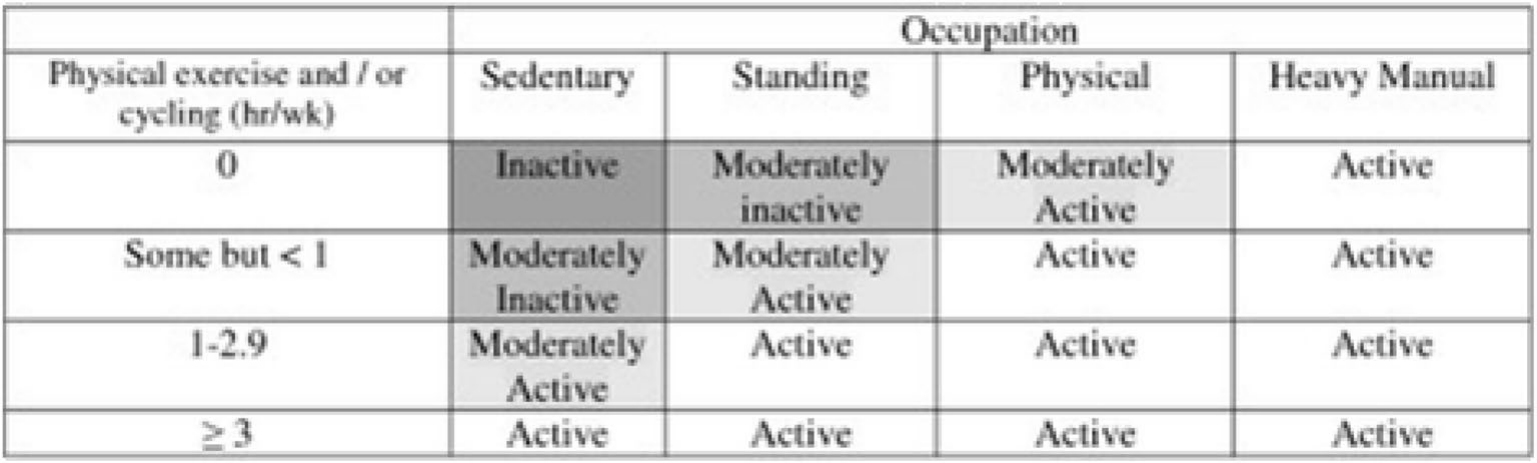
Summary of the GPPAQ Physical Activity Index Scoring.

Between 2020 and 2022, this case–control study included adults over the age of 18 who had recently been diagnosed with NAFLD and healthy controls who had been admitted to Taleghani Hospital in Tehran, Iran, and the academic liver disease clinics of Shahid Sadoughi University of Medical Sciences in Yazd, Iran. The control group included 552 people without a history of NAFLD who were recruited from the same hospital, whereas the case group included 340 consecutive patients with NAFLD who had been diagnosed by a gastroenterologist. The procedure for patient sample was assessed by two dietitians. The following criteria were used to diagnose NAFLD^28–30^: chronic elevation in liver enzymes (liver enzymes > 19 U/L for women and > 30 U/L for men), liver ultrasound compatible with NAFLD, Having a grade II, III NAFLD based on liver biopsy, abstinence from alcohol usage, and the elimination of other possible causes of liver disease. A gastroenterologist confirmed the diagnosis of NAFLD when the case group was submitted to our facilities for assessment by Fibroscan^28^, which indicated a controlled attenuation parameter score of more than 237 and a fibrosis score of more than 7. Also, patients from other outpatient clinics at the same hospital, including dermatology, ophthalmology, and otorhinolaryngology, were recruited for the control group, which had no history of NAFLD. The healthy controls had no history of chronic or inflammatory illnesses, had been eating consistently for the past six months, and had been physically active (such as diabetes, gastrointestinal or cardiovascular disorders, cancer, etc.). Laboratory tests and liver ultrasonography, which confirmed they were free of any hepatic steatosis in any stage, served as the foundation for the inclusion criteria for the control group. Also, matching of people in the case and control group (1:1) was done based on age variables (± 3 years) and body mass index (BMI) (± 1 kg/m2). The following conditions precluded patients from participating: long-term dietary changes, weight loss, a specific illness, a history of hepatic or renal disease (such as non-alcoholic steatohepatitis (NASH), alcoholic fatty liver disease, Wilson's disease, cirrhosis, autoimmune liver disease, hemochromatosis, viral infections), diabetes, cancer, thyroid disorder, and autoimmune disease. By completing demographic, economic, and social questionnaires, information regarding age, education level, work status, medical history, smoking status, usage of certain pharmaceuticals (other than typical NAFLD medications), and dieting history during the past six months was gathered. The levels of physical activity of the participants were assessed using general practice physical activity questionnaires (GPPAQs). The GPPAQ is a short survey that gauges one's current level of physical activity^30^ and occupation and is scored into active, moderately active, moderately inactive or inactive categories (see Fig. 1). In this study, nutritionists served as the interviewers. As a consequence, every patient answered every item in the survey honestly. Also, informed consent was obtained from all subjects and all methods were performed in accordance with the relevant guidelines and regulations. This study was approved by Shahid Beheshti University of Medical Sciences, Tehran, Iran, and Shahid Sadoughi University of Medical Sciences, Yazd, Iran.

### Calculation of sample size

Required minimal sample size for the current work was calculated based on the hypothesis of 1.5 times decreased odds of NAFLD by OBS. Terefore, considering type I error of 5%, the study power of 90% and the ratio of controls to cases as approximately 1.5, the minimum required sample size was calculated.

### Anthropometric measurement

The researchers carried out an anthropometric analysis. The weight was recorded to the closest 100 g using a standard SECA 700 Digital Scale (SECA, Hamburg, Germany) with little clothing and no shoes. The patient's height requirements were evaluated using a Seca portable height gauge with 0.1 cm of precision. In addition, a Seca waist measuring device was used to establish the waist circumference (WC) in the central area between the iliac crown and the last gear. In addition, the hip circumference was calculated in centimeters by placing the same measuring tape parallel to the floor at the fullest part of the buttocks. Weight (kg)/Height^2^(m) was used to determine body mass index (BMI) after weight and height were measured in the aforementioned method. All anthropometric measures were conducted by the researcher in order to reduce observational variance.

### Dietary assessment

A semi-quantitative validated food-frequency questionnaire (FFQ) with 168 food items was used to collect data on dietary consumption during the preceding year^31^. The FFQ consisted of a list of typical Iranian foods and their serving sizes. Self-reports on the FFQ determined the average portion size and frequency of consumption for each food item. The frequency of consumption of each food item was as follows: never, 2–3 times per month, once per week, 2–4 times per week, 5–6 times per week, and daily. Using standard Iranian household measurements, the serving quantities were provided in grams^32^. Utilizing the United States Department of Agriculture's (USDA) national nutritional databank, daily nutrient consumptions for each individual were calculated^33^. The nutritional and calorie content of the foods were analyzed using a customized version of Nutritionist 4 (First DatabankInc., Hearst Corp., San Bruno, CA, USA) for Iranian meals.

### Calculation of oxidative balance scores (OBS)

In the present study, we used the method described by Goodman et al.^34^ to calculate the OBS of each participant. According to this method, a total of 13 dietary and nondietary pro- and antioxidant components, based on a priori knowledge about their association to oxidative stress, were selected. The components were divided into four groups: (1) dietary antioxidants (selenium, fiber, β-carotene, vitamin D, vitamin C, vitamin E, and folate); (2) dietary prooxidants (iron, saturated (SFA), and polyunsaturated (PUFA) fatty acids); (3) nondietary antioxidant (physical activity); and (4) nondietary prooxidants (smoking and obesity). Dietary factors were ranked into quintiles. For dietary antioxidants and physical activity, the first to fifth quintiles were assigned scores of 1–5. An inverse scoring was used for dietary prooxidants. For obesity, we assigned 1: BMI ≥ 30 kg/m^2^ and WC ≥ 102 cm in males and ≥ 88 cm in females, 3: either BMI ≥ 30 kg/m^2^ or WC ≥ 102 cm in males or ≥ 88 cm in females, and 5: BMI < 30 kg/m^2^ and WC < 102 in males or < 88 cm in females. For smoking, it was assigned 1: current smoking, 3: former smoking, and 5: never smoking. The score of four components was then summed to calculate the OBS for each participant. A higher score of OBS indicates more adherence to this score derived from diet and a lower score indicates less adherence to this score. The minimum and maximum scores possible are, respectively, 5 and 65.

### Biochemical measurement:

The laboratory technician took 10 ml of venous blood from the individuals at the beginning and end of the trial, following 10–12 h of fasting. After clotting in the environment, the serum was quickly separated by centrifugation and stored at −70 °C until it was transported to the laboratory for testing. The concentrations of triglycerides (TG), high-density lipoprotein cholesterol (HDL-C), and fasting blood sugare (FBS) were determined using a kit from Pars Azmon Company (Tehran, Iran) using an enzymatic colorimetric technique. Enzyme photometry was used with the Pars test kit (Parsazmun, Tehran, Iran) to determine the total cholesterol content. Using the Friedewald formula^35^, the concentration of low-density lipoprotein cholesterol (LDL-C) was also determined. LDL-C = TC (mg/dL) − HDL-C (mg/dL) − TG (mg/dL)/5. On the basis of an auto analysis (BT-3000)., the enzymes alanine aminotransferase (ALT) and aspartate aminotransferase (AST) were measured using commercially available enzymatic reagents (Pars Azmoon, Tehran, Iran).

### Statistical analysis

The Statistical Package Software for Social Science v.21 (SPSS Inc., Chicago, IL, USA) was used to conduct the statistical analysis. The data's normality was examined using the Kolmogorov–Smirnov’s test and histogram charts. The baseline characteristics and dietary intakes were recorded as mean standard deviation (SD) for quantitative variables, and for qualitative variables, as number and percentages. We used independent sample t-tests (or one-way ANOVA) and chi-squared tests to compare data between two groups (or across quartiles of OBS) for continuous and categorical variables, respectively. Nutrients were adjusted for total energy intake (kcal) using the residual method. Logistic regression was used to examine the relationship between OBS and the risk of NAFLD. The analyses were adjusted for potential confounders such as age, sex, hip circumference, education, drug use, disease history, FBS, ALT, AST, Lipid profiles, and energy intake. The odds ratio (OR) of NAFLD across quartiles of scores was estimated with a 95% confidence interval (CI). P-values < 0.05 were considered statistically significant.

### Ethics approval and consent to participate

This study was approved by the research council and ethics committee Shahid Beheshti University of Medical Sciences, Tehran, Iran.

## Results

The mean (± SD) age of the study population was 40.22 ± 9.79 years. The mean (± SD) BMI was 29.06 ± 3.92 kg/m^2^. The mean ± SD of OBS was 41.48 ± 5.23.

Table 1 illustrates the general characteristics and biochemical parameters of participants between NAFLD patients and control groups as well as across the quartile of OBS. Compared with controls, NAFLD subjects had significantly higher hip circumference, ALT, AST, FBS, TC, TG, and LDL-C concentration, but had lower physical activity and mean of OBS. There was also a significant difference between the level of education, diseases history, drug use, and smoking between the case and control groups. The mean age of subjects in the highest quartile of OBS vs the lowest quartile, significantly decreased. Also, the mean BMI, weight, WC, Hip- circumference, as well as use of smoking, drug, and disease history decreased across the quartile of OBS, but physical activity increased. In addition, a significant difference was observed between the levels of education among the quartile of OBS. There were no significant differences between the quartile of OBS and rest of variables.

**Table 1:** Socio-demographic characteristics and anthropometric variables between groups and across the Quartiles of oxidative balance score (OBS).

	Groups		P-value^a^	Quartiles of OBS				P-value^b^
	Case (N = 552)	Control (N = 340)		Q1 [25–37] (N = 223)	Q2 [38–41] (N = 222)	Q3 [42–45] (N = 223)	Q4 [46–60] (N = 223)	
Demographic variables								
Age, years	40.52 (9.65)	39.92 (9.83)	0.459	41.13 (10.22)	40.38 (10.38)	38.84 (9.36)	37.74 (8.79)	**0.002**
Female, n (%)	190 (55.9)	290 (52.5)	0.330	115 (58.4)	133 (54.5)	136 (54.6)	96 (47.8)	0.193
BMI^b^, kg/m^2^	29.50 (4.04)	28.62 (3.81)	0.119	28.56 (4.58)	27.54 (4.49)	26.66 (4.39)	25.70 (3.82)	** < 0.001**
Weight, kg	81.74 (12.83)	79.98 (11.51)	0.589	77.89 (13.68)	74.22 (15.13)	72.03 (12.79)	67.23 (10.79)	** < 0.001**
Waist-circumference (cm)	99.45 (8.59)	96.98 (7.97)	0.712	98.64 (10.79)	93.16 (12.11)	91.65 (11.54)	86.87 (9.13)	** < 0.001**
Hip-circumference (cm)	103.00 (9.52)	99.16 (6.85)	**0.045**	105.53 (9.92)	101.92 (9.10)	100.16 (8.140	97.68 (7.69)	** < 0.001**
Physical activity (Met.h/wk)	1119.03 (616.35)	1590.30 (949.44)	** < 0.001**	952.45 (441.52)	1235.48 (764.93)	1417.68 (856.98)	1859.72 (976.89)	** < 0.001**
Smoking (yes), n (%)	16 (7.1)	12 (2.7)	**0.006**	10 (10.4)	9 (5.4)	9 (4.1)	0 (0.0)	** < 0.001**
Disease history (yes), n (%)	45 (13.2)	37 (6.7)	**0.001**	34 (17.3)	28 (11.5)	15 (6.0)	4 (2.0)	** < 0.001**
Drug use (yes), n (%)	55 (20.4)	30 (6.4)	** < 0.001**	34 (27.0)	30 (15.6)	17 (7.5)	4 (2.1)	** < 0.001**
Education n (%)								
Less than a diploma	77 (22.6)	83 (15)	**0.001**	51 (25.9)	48 (19.7)	36 (14.5)	24 (11.9)	** < 0.001**
Diploma	124 (36.5)	208 (37.7)		75 (38.1)	82 (33.6)	88 (35.3)	87 (43.3)	
Bachelor	99 (29.1)	148 (26.8)		33 (16.8)	69 (28.3)	83 (33.3)	62 (0.8)	
Higher than Bachelor	40 (11.8)	113 (20.5)		38 (19.3)	45 (18.4)	42 (16.9)	28 (13.9)	
ALT(mg/dl)	42.13 (34.78)	19.75 (8.61)	** < 0.001**	34.17 (22.25)	36.58 (52.15)	36.63 (39.12)	26.09 (38.18)	0.745
AST(mg/dl)	28.59 (14.90)	17.58 (5.51)	** < 0.001**	30.86 (24.14)	32.87 (35.30)	32.03 (22.97)	26.71 (27.48)	0.935
FBS (mg/dl)	120.76 (47.74)	107.14 (26.07)	**0.013**	113.89 (37.41)	119.47 (46.92)	106.45 (28.73)	96.00 (3.51)	0.264
TC (mg/dl)	185.21 (46.73)	165.42 (44.86)	**0.002**	177.62 (48.61)	178.85 (46.56)	196.19 (43.60)	162.14 (42.81)	0.613
TG (mg/dl)	200.51 (89.62)	141.07 (69.00)	** < 0.001**	180.94 (90.82)	169.02 (86.41)	169.16 (71.78)	112.28 (41.55)	0.208
LDL-C (mg/dl)	114.59 (35.77)	103.44 (34.83)	**0.023**	106.86 (36.98)	110.18 (34.41)	116.61 (36.64)	101.85 (27.20)	0.553
HDL-C (mg/dl)	42.08 (9.34)	45.73 (18.07)	**0.061**	43.51 (15.15)	42.45 (8.68)	46.90 (20.87)	48.71 (9.21)	0.389
Mean of OBS	39.72 (4.81)	42.56 (5.19)	** < 0.001**	34.37 (2.31)	39.47 (1.08)	43.42 (1.15)	48.47 (2.65)	< 0.001

Values are expressed as means (standard deviation (SD)) of 892 subjects.

Significant values are in bold.

P-values are resulted from one way-ANOVA^b^ Test or independent sample T-Test^a^ for continuous variables and Chi-square for categorical variables.

*BMI* body mass index, *FBS* fasting blood sugar, *HDL-C* high density lipoprotein-cholesterol, *LDL-C* low density lipoprotein-cholesterol, *TC* total cholesterol, *TG* triglycerides, *AST* aspartate aminotransferase, *ALT* alanine transaminase.

Dietary intake of subjects between groups and across the quartile of OBS are presented in Table 2. NAFLD subjects had higher intakes of energy, protein, saturated fatty acid (SFA), and red and processed meats, but lower intakes of calcium, magnesium, folate, vitamin D, legume, whole grain, fruits, and vegetables as compared to controls. Compared with those in the lowest quartile of OBS, subjects in the highest quartile had higher intake of energy, carbohydrate, fiber, calcium, magnesium, zinc, vitamin C, E, D, B9, total dairy, whole grains, fruits, and vegetables as well as lower intake of protein, fat, monounsaturated fatty acid (MUFA), polyunsaturated fatty acid (PUFA), cholesterol, sodium, iron, nuts, and red and processed meat.

**Table 2:** Dietary intake between groups and across the Quartiles of oxidative balance score (OBS).

	Groups		P-value^a^	Quartiles of OBS				P-value^b^
	Case (N = 552)	Control (N = 340)		Q1 [25–37] (N = 223)	Q2 [38–41] (N = 222)	Q3 [42–45] (N = 223)	Q4 [46–60] (N = 223)	
Dietary intake								
Energy (Kcal/day)	2301.47 (628.15)	2148.31 (645.50)	**0.001**	1953.88 (597.83)	2035.62 (628.72)	2358.02 (628.08)	2469.56 (565.57)	** < 0.001**
Carbohydrate (g/day)	317.77 (46.23)	315.48 (40.49)	0.437	301.97 (39.23)	316.21 (40.84)	317.99 (42.38)	328.82 (44.83)	** < 0.001**
Protein (g/day)	81.25 (20.20)	77.75 (15.92)	**0.004**	85.60 (21.21)	78.59 (18.35)	76.72 (15.36)	76.10 (14.08)	** < 0.001**
Fat (g/day)	76.07 (19.41)	77.57 (16.39)	0.216	81.41 (14.89)	77.13 (17.66)	76.63 (18.32)	72.90 (18.23)	** < 0.001**
SFA (g/day)	25.81 (7.14)	24.29 (7.79)	**0.003**	26.08 (7.29)	24.77 (6.86)	25.34 (7.91)	24.84 (7.59)	0.249
MUFA	25.38 (8.06)	26.18 (6.55)	0.107	26.84 (6.07)	25.84 (7.58)	26.07 (7.47)	24.78 (7.17)	**0.039**
PUFA	17.26 (7.92)	16.73 (6.75)	0.287	19.59 (8.26)	17.14 (6.37)	16.30 (6.51)	14.92 (7.14)	** < 0.001**
Cholesterol (mg/day)	247.47 (128.43)	245.00 (129.48)	0.781	281.47 (125.34)	250.98 (169.14)	229.39 (102.25)	224.50 (93.50)	** < 0.001**
Fiber (g/day)	32.86 (19.03)	33.50 (14.63)	0.577	23.76 (10.79)	32.38 (18.00)	35.92 (16.14)	40.39 (14.90)	** < 0.001**
Sodium (mg/day)	3896.65 (3445.74)	4202.41 (2779.62)	0.146	4089.10 (2743.28)	4723.47 (4202.28)	3834.67 (2661.78)	3592.86 (1643.55)	** < 0.001**
Iron (mg/day)	35.41 (58.57)	29.68 (28.66)	0.051	40.97 (58.87)	29.71 (40.44)	30.27 (44.15)	27.41 (14.07)	**0.007**
Calcium (mg/day)	1054.73 (381.25)	1118.62 (357.33)	**0.012**	937.19 (324.59)	1012.93 (326.26)	1156.04 (363.61)	1269.51 (371.15)	** < 0.001**
Magnesium (mg/day/)	333.62 (75.86)	346.57 (79.41)	**0.016**	299.45 (58.22)	325.68 (62.29)	357.37 (84.11)	383.25 (79.69)	** < 0.001**
Zinc (mg/day)	10.72 (2.17)	10.83 (2.05)	0.451	10.64 (1.81)	10.42 (1.79)	10.96 (2.38)	11.15 (2.25)	**0.001**
Vitamin C (mg/day)	156.79 (90.07)	147.59 (80.06)	0.113	131.02 (56.68)	141.44 (77.48)	150.82 (95.19)	181.70 (90.07)	** < 0.001**
Folate (mcg/day)	446.97 (150.87)	473.74 (118.14)	**0.003**	371.75 (120.13)	449.75 (131.83)	491.54 (118.25)	536.62 (100.42)	** < 0.001**
Vitamin E (mg/day)	10.35 (4.30)	10.73 (4.33)	0.201	9.42 (4.03)	10.22 (4.13)	11.18 (4.16)	11.46 (4.71)	** < 0.001**
Vitamin D (mcg/day)	1.45 (1.26)	1.88 (1.67)	** < 0.001**	1.03 (0.78)	1.38 (1.08)	1.90 (1.53)	2.56 (2.09)	** < 0.001**
Caffeine (mg/day)	126.15 (113.48)	128.60 (105.80)	0.782	116.10 (74.13)	123.76 (73.99)	126.39 (120.57)	138.62 (130.68)	0.345
Food groups								
Total dairy (g/day)	378.99 (244.72)	385.46 (224.39)	0.686	308.68 (215.43)	351.06 (208.41)	413.41 (237.05)	457.13 (242.83)	** < 0.001**
Legume (g/day)	18.95 (19.18)	22.03 (26.54)	**0.046**	20.95 (14.97)	21.86 (23.57)	18.61 (24.23)	19.04 (24.20)	0.337
Nut (g/day)	8.73 (11.77)	8.25 (10.88)	0.539	11.70 (11.91)	8.40 (9.62)	7.32 (11.71)	6.69 (11.14)	** < 0.001**
Fish (g/day)	10.34 (9.81)	10.52 (14.57)	0.842	11.77 (20.24)	10.64 (10.95)	9.41 (9.30)	10.21 (9.50)	0.291
Whole grains (g/day)	75.08 (71.7)	88.45 (89.81)	**0.020**	69.01 (45.88)	86.47 (71.98)	93.87 (111.34)	81.13 (83.62)	**0.016**
Refined grains (g/day)	308.01 (193.93)	290.74 (146.27)	0.132	276.83 (132.73)	308.89 (178.09)	301.53 (174.26)	298.93 (169.78)	**0.226**
Red and Processed meat (g/day)	39.94 (34.05)	31.45 (28.42)	** < 0.001**	50.03 (37.31)	35.89 (28.65)	29.35 (26.85)	24.70 (25.31)	** < 0.001**
Fruits (g/day)	420.43 (301.10)	480.76 (366.12)	**0.008**	412.50 (254.43)	438.77 (312.62)	422.46 (383.03)	501.10 (328.89)	**0.028**
Vegetables (g/day)	269.38 (158.26)	302.36 (149.91)	**0.002**	259.78 (131.18)	283.68 (156.73)	285.57 (155.29)	332.02 (161.69)	** < 0.001**

Values are expressed as means (standard deviation (SD)) of 892 subjects. Nutrients were adjusted for total energy intake (kcal).

Significant values are in bold.

P-values are resulted from one way-ANOVA^b^ Test or independent sample T-Test^a^ for continuous variables.

*PUFA* polyunsaturated fatty acid, *SFA* saturated fatty acid, *MUFA* monounsaturated fatty acid.

The ORs and 95% CIs for NAFLD subjects based on quartile of OBS of are reported in Table 3.

**Table 3:** Odds ratio (OR) and 95% confidence interval (CI) for NAFLD based on oxidative balance score (OBS).

	Quartiles of OBS				P for trend
	Q1	Q2	Q3	Q4	
OBS score					
Case/Control	107/116	99/123	94/129	39/184	
Crude model	1.00 (Ref)	0.40 (0.25–0.64)	0.41 (0.26–0.64)	0.35 (0.21–0.56)	** < 0.001**
Model 1*	1.00 (Ref)	0.41 (0.26–0.66)	0.43 (0.27–0.67)	0.38 (0.26–0.63)	** < 0.001**
Model 2^†^	1.00 (Ref)	0.35 (0.21–0.59)	0.31 (0.18–0.52)	0.29 (0.15–0.49)	** < 0.001**

Significant values are in bold.

**Binary logistic regression was used to obtain OR, 95% CI and P for trend.

*Model 1: adjusted for age and sex.

^†^Model 2: Model 1 + hip circumference, education, drug use, disease history, FBS, ALT, AST, Lipid profiles, and energy intake.

In crude and first adjusted model (based on age and sex), there was a significant association for OBS in the highest quartile compared with the lowest quartile (odds ratio [OR] = 0.35, 95% confidence interval [CI] 0.21–0.56; P for trend < 0.001; OR = 0.38, 95% CI 0.26–0.63; P for trend < 0.001, respectively). Furthermore, after adjusting for confounders according to the final model (based on model 1 + hip circumference, education, drug use, disease history, FBS, ALT, AST, Lipid profiles, and energy intake), higher scores of adherence to the OBS conferred a protection for the presence of NAFLD (OR: 0.29; 95% CI 0.15–0.49; P for trend < 0.001).

## Discussion

This study examined the link between an OBS and NAFLD risk in 340 NAFLD patients and 552 controls, all of whom were men and women over the age of 18 who visited a hospital in Tehran or Yazd, Iran. We used a previously used score in other studies that is based on a remarkable compilation of 13 components that extensively assess exposure through dietary and lifestyle-related antioxidant and pro-oxidant components, allowing us to approach the complexity of individual oxidative balance assessment comprehensively. After controlling for relevant confounders, our data revealed a statistically significant strong inverse relationship between OBS score and decreased NAFLD risk. So that the findings show a protective effect on NAFLD following OBS adherence.

To our knowledge, this is the first study to investigate the OBS as a protective factor against NAFLD. However, several studies have investigated the potential beneficial effects of this index on other chronic diseases such as metabolic syndrome^36^, diabetes^37^, cancer^38^, kidney^39,40^, and cardiovascular diseases^40^ which seemed to have a similar mechanism to the risk of NAFLD. In addition, the relationship between this index with the reduction of oxidative stress and systemic inflammation has been investigated as a major risk factor in NAFLD^41^. So that a study in 2019 by Golmohammadi et al^37^ showed that higher compliance with OBS, which indicates increased exposure to antioxidants and decreased exposure to prooxidants, improved insulin resistance as well as better glycemic control in adults with type 2 diabetes. A cross-sectional study conducted among 6,400 Korean adults over 40 years of age showed that compared to the lowest tertile, those in the highest tertile of OBS had a 35% lower risk of developing metabolic syndrome after adjustment for potential confounders^36^. In addition, in a study, increased adherence to OBS was associated with a decreased incidence of end-stage renal disease (ESRD)^39^. However, in another study, no significant relationship between this index and the incidence of ESRD and cardiovascular disease was observed after adjustment of confounders^40^, which according to the author's statement could be due to the specific dietary restrictions of these patients at the time of study entry.

In a study by Kong et al.^41^, the relationship between OBS and markers of oxidative stress and inflammation was investigated. The findings of this study indicated that people with higher OBS adherence had a decrease in the levels of F2-isoprostanes (FIP) and C-reactive protein (CRP) by 80% and 40%, respectivcely, compared to people with lower adherence to this index.

Furthermore, evidence shows that micronutrients with antioxidant, antifibrotic, immunomodulatory, and lipoprotective capabilities, such as vitamins A, C, D, and E, carotenoids, zinc, selenium, and magnesium, may have favorable effects on NAFLD^42^. On the other hand, the findings of the present case–control study are consistent with recent studies and evidence on the protective role of healthy dietary patterns. These dietary patterns, characterized by high intake of fruits, vegetables, legumes, and low-fat dairy products, which are rich in antioxidant nutrients and with minimal prooxidants, can reduce the incidence of NAFLD^43^. The formation and progression of NAFLD are both influenced by poor eating, which is defined as a diet high in calories, carbohydrates, and saturated fats and lacking in fiber, MUFA, and antioxidant micronutrients^44^. Additionally, a systematic review and meta-analysis of the available data showed that western dietary patterns with high intakes of processed foods, red meat, high-fat dairy products, and refined grains with minimal intake of antioxidant nutrients and rich in prooxidants might considerably increase the likelihood of developing NAFLD (OR 1.56; 95% CI 1.27–1.92). Furthermore, research revealed that cooking beef at high temperatures for an extended period of time produces heterocyclic amines (HCAs), which are linked to oxidative stress and NAFLD^45^.

It is also important to note that lifestyle as one of the components of OBS can also play an important role in the incidence of NAFLD in our study. This suggests that each of the healthy lifestyle variables, such as physical exercise, an appropriate BMI, and quitting smoking, played a significant effect in avoiding NAFLD in addition to nutrition. Obesity, a sedentary lifestyle, smoking, as well as other individual variables and environmental factors, have been demonstrated to impact NAFLD^46^.

The study offers both advantages and disadvantages. One of the study's shortcomings is that it is unable to evaluate the causal connection. Another drawback is that, despite accounting for a number of potential confounding factors, it is impossible to rule out the chance that any more potential confounding factors may exist that were not taken into account in the studies. Self-reported data on food consumption might also lead to memory bias. Another weakness of this study may be that because the subjects were Muslims, alcohol intake was not examined. A review of this OBS component, though, would have no impact on the outcomes because neither the control group nor the study case group drank alcohol.

The recent research has a number of advantages. This is the first study that we are aware of that evaluated the relationship between OBS and the risk of NAFLD in Iranian adults. Trained workers were used to conduct interviews and gather food frequency questionnaires. The sample size we used was adequate, and we made an effort to account for a variety of confounding factors by utilizing a validated questionnaire and large-scale variable adjustments.

*In conclusion*, by calculating the study power of approximately 90%, the findings of the present study indicate an approximately 80% reduction in the odds of developing NAFLD with higher OBS adherence. Our findings confirm earlier research on the prevention of NAFLD by good food and lifestyle choices. Additionally, they can be used as tactics to stop or even slow the progression of NAFLD.

## Data Availability

Data is available upon request from the corresponding author for the article due to privacy/ethical restrictions.
